# (2*S*)-Methyl 2-(*p*-toluenesulfonamido)propanoate

**DOI:** 10.1107/S1600536809017371

**Published:** 2009-05-14

**Authors:** Tayyaba Syed, Shahid Hameed, Peter G. Jones, Andrea Schmidt-Meier

**Affiliations:** a Department of Chemistry, Quaid-i-Azam University, Islamabad-45320, Pakistan; b Institut for Anorganische und Analytische Chemie, Technische Universität Braunschweig, Hagenring 30, 38106 Braunschweig, Germany

## Abstract

The enanti­omerically pure title compound, C_11_H_15_NO_4_S, contains a pyramidal N atom with an S—N bond length of 1.6262 (8) Å. In the crystal, mol­ecules are linked to form chains parallel to the **a** axis by the hydrogen bond from NH to the carbonyl oxygen. C—H⋯O inter­actions are also present.

## Related literature

For the applications of esters in the food and cosmetics industries, see: Soni *et al.* (2002 ▶). For their use as inter­mediates in the synthesis of heterocyclic compounds, see: Akhtar *et al.* (2007 ▶); Chen *et al.* (2007 ▶); Kucukguzel *et al.* (2007 ▶). For their use in the pharmaceutical industry, see: Iqbal & Chaudhry (2008 ▶). For the pharmacological activity of sulfonamides, see:Akhtar *et al.* (2008 ▶); Zareef *et al.* (2007 ▶). For a description of the Cambridge Structural Database, see: Allen (2002 ▶).
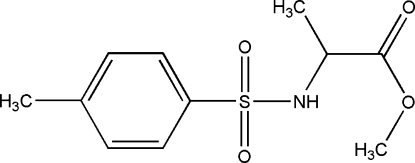


## Experimental

### 

#### Crystal data

C_11_H_15_NO_4_S
*M* *_r_* = 257.30
Orthorhombic, 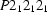

*a* = 7.1948 (2) Å
*b* = 11.2552 (3) Å
*c* = 15.3311 (4) Å
*V* = 1241.50 (6) Å^3^
*Z* = 4
Mo *K*α radiation
μ = 0.26 mm^−1^
*T* = 100 K
0.35 × 0.30 × 0.20 mm

#### Data collection

Oxford Diffraction Xcalibur E diffractometer
Absorption correction: multi-scan (*CrysAlis RED*; Oxford Diffraction, 2008 ▶) *T*_min_ = 0.972, *T*_max_ = 1.000 (expected range = 0.922–0.949)
55416 measured reflections
4284 independent reflections
4060 reflections with *I* > 2σ(*I*)
*R*_int_ = 0.028

#### Refinement

*R*[*F* ^2^ > 2σ(*F* ^2^)] = 0.022
*wR*(*F* ^2^) = 0.063
*S* = 1.05
4284 reflections
161 parameters
H atoms treated by a mixture of independent and constrained refinement
Δρ_max_ = 0.37 e Å^−3^
Δρ_min_ = −0.31 e Å^−3^
Absolute structure: Flack (1983 ▶), 1818 Friedel pairs
Flack parameter: 0.01 (4)

### 

Data collection: *CrysAlis CCD* (Oxford Diffraction, 2008 ▶); cell refinement: *CrysAlis RED* (Oxford Diffraction, 2008 ▶); data reduction: *CrysAlis RED*; program(s) used to solve structure: *SHELXS97* (Sheldrick, 2008 ▶); program(s) used to refine structure: *SHELXL97* (Sheldrick, 2008 ▶); molecular graphics: *XP* (Siemens, 1994 ▶); software used to prepare material for publication: *SHELXL97*.

## Supplementary Material

Crystal structure: contains datablocks I, global. DOI: 10.1107/S1600536809017371/hg2507sup1.cif

Structure factors: contains datablocks I. DOI: 10.1107/S1600536809017371/hg2507Isup2.hkl

Additional supplementary materials:  crystallographic information; 3D view; checkCIF report

## Figures and Tables

**Table 1:** Hydrogen-bond geometry (Å, °)

*D*—H⋯*A*	*D*—H	H⋯*A*	*D*⋯*A*	*D*—H⋯*A*
N—H01⋯O1^i^	0.865 (16)	2.057 (16)	2.9097 (10)	168.6 (14)
C10—H10⋯O1^i^	0.95	2.62	3.3696 (11)	136
C9—H9⋯O2^ii^	0.95	2.63	3.4065 (11)	140
C7—H7⋯O3^iii^	0.95	2.67	3.6167 (11)	172
C4—H4*C*⋯O4^iv^	0.98	2.49	3.3453 (13)	145

Symmetry codes: (i) 
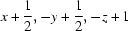
; (ii) 
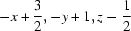
; (iii) 
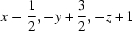
; (iv) 
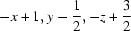
.

**Fig. 1.**
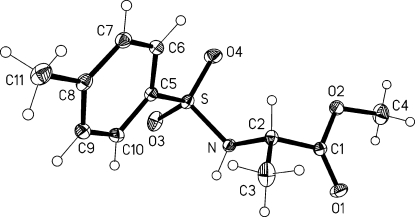
The molecule of the title compound. Ellipsoids correspond to 50% probability levels.

**Fig. 2.**
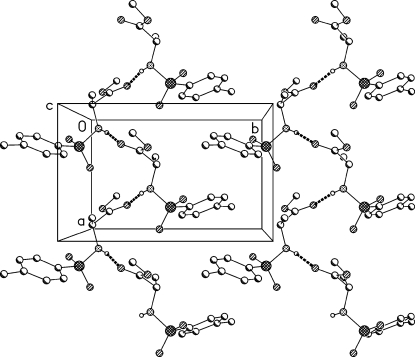
Packing diagram of the title compound, showing classical hydrogen bonds (dashed lines).

## supplementary crystallographic information

### Comment

Because of their versatility and harmlessness, esters have found many applications in the food and cosmetics industries (Soni *et al.*, 2002). They have been reported as emulsifiers, dispersants, or thickeners. In addition to their use as intermediates in the synthesis of a large number of heterocyclic compounds (Chen *et al.*, 2007; Kucukguzel *et al.*, 2007; Akhtar *et al.*, 2007), esters have also been used in the pharmaceutical industry as nervous system depressants, intestinal antiseptics and antibacterials (Iqbal *et al.*, 2008). Sulfonamides, on the other hand, constitute an important class of drugs with several types of pharmacological activities (Akhtar *et al.*, 2008; Zareef *et al.*, 2007). The title compound (I) was synthesized in our laboratory as an intermediate for onward conversion to 1,3,4-oxadiazole derivatives, with a view to explore their anti-HIV and anti-HCV activities.

The molecule of (I) is shown in Fig. 1. Bond lengths and angles may be regarded as normal. In particular, the N—S bond length is 1.6262 (8) Å; a search of the Cambridge Database (Allen, 2002; Version 1.11) revealed 817 examples of the fragment Ph—SO_2_—NH—C(*sp*^3^) (including substituted Ph rings) with a mean bond length of 1.614 Å. The nitrogen atom displays a pyramidal geometry, with N lying 0.293 (7) Å out of the plane of S, C2 and H01. The database hits showed a highly skewed distribution for this displacement, with many values of exactly zero; this is presumably attributable to fixing the hydrogen assuming planar geometry (AFIX 43 in the *SHELX* system), whereby the assumption of planarity is clearly not justified. Neglecting the zero values, the average deviation for 462 values is 0.24 Å. The sulfonyl group is oriented such that the bond S—O4 is approximately coplanar with the aromatic ring (torsion angle O4—S—C5—C6 - 6.70 (9)°).

The molecules are linked by classical hydrogen bonds from the NH group to the carbonyl oxygen (and not, as might have been expected, to a sulfonyl oxygen); the packing diagram (Fig. 2) shows chains of molecules parallel to the a axis. The four "weak" hydrogen bonds crosslink these chains to a three-dimensional pattern.

### Experimental

Alanine (0.02 mol) was dissolved in an aqueous solution of potassium carbonate (0.06 mol, 50 ml) and a solution of 4-methylbenzenesulfonyl chloride (0.027 mol) in toluene (30 ml) was added. On completion of the reaction, the organic layer was separated and the aqueous layer acidified using dilute hydrochloric acid. The 2-(4-methylphenylsulfonylamino)propanoic acid thus obtained was filtered off and recrystallized from aqueous ethanol.

2-(4-Methylphenylsulfonylamino)propanoic acid (0.02 mol) was dissolved in methanol (30 ml), sulfuric acid (0.5 ml) was added and the mixture subjected to reflux while the reaction was monitored by TLC at regular intervals. After completion of the reaction, the reaction mixture was concentrated on the rotary evaporator to remove excess methanol. The product thus obtained was poured into water, neutralized with sodium bicarbonate and extracted with ethyl acetate (3 × 50 ml). The combined organic extracts were dried over anhydrous sodium sulfate and the solvent evaporated on a rotary evaporator. The product was recrystallized from acetone/water.

### Refinement

The NH hydrogen was refined freely. Methyl H atoms were located in difference syntheses, idealized to C—H 0.98 Å and H—C—H 109.5°, and refined as rigid groups allowed to rotate but not tip. Other H atoms were placed in calculated positions and refined using a riding model with C—H 0.95 Å for aromatic H and 1.00 Å for methine CH. Hydrogen U values were fixed at 1.5 × U(eq) of the parent atom for methyl H and 1.2 × U(eq) of the parent atom for other H. The compound is enantiomerically pure and its absolute configuration (S at C2) was confirmed by the Flack (1983) parameter.

### Crystal data

**Table:**

C_11_H_15_NO_4_S	*D*_x_ = 1.377 Mg m^−^^3^
*M**_r_* = 257.30	Melting point = 363–365 K
Orthorhombic, *P*2_1_2_1_2_1_	Mo *K*α radiation, λ = 0.71073 Å
Hall symbol: P 2ac 2ab	Cell parameters from 40101 reflections
*a* = 7.1948 (2) Å	θ = 2.2–32.6°
*b* = 11.2552 (3) Å	µ = 0.26 mm^−^^1^
*c* = 15.3311 (4) Å	*T* = 100 K
*V* = 1241.50 (6) Å^3^	Irregular block, colourless
*Z* = 4	0.35 × 0.30 × 0.20 mm
*F*(000) = 544	

### Data collection

**Table:**

Oxford Diffraction Xcalibur E diffractometer	4284 independent reflections
Radiation source: fine-focus sealed tube	4060 reflections with *I* > 2σ(*I*)
graphite	*R*_int_ = 0.028
Detector resolution: 16.1419 pixels mm^-1^	θ_max_ = 32.0°, θ_min_ = 2.2°
ω–scan	*h* = −10→10
Absorption correction: multi-scan (*CrysAlis RED*; Oxford Diffraction, 2008)	*k* = −16→16
*T*_min_ = 0.972, *T*_max_ = 1.000	*l* = −22→22
55416 measured reflections	

### Refinement

**Table:**

Refinement on *F*^2^	Secondary atom site location: difference Fourier map
Least-squares matrix: full	Hydrogen site location: inferred from neighbouring sites
*R*[*F*^2^ > 2σ(*F*^2^)] = 0.022	H atoms treated by a mixture of independent and constrained refinement
*wR*(*F*^2^) = 0.063	*w* = 1/[σ^2^(*F*_o_^2^) + (0.0451*P*)^2^ + 0.0628*P*] where *P* = (*F*_o_^2^ + 2*F*_c_^2^)/3
*S* = 1.05	(Δ/σ)_max_ = 0.001
4284 reflections	Δρ_max_ = 0.37 e Å^−^^3^
161 parameters	Δρ_min_ = −0.31 e Å^−^^3^
0 restraints	Absolute structure: Flack (1983), 1818 Friedel pairs
Primary atom site location: structure-invariant direct methods	Flack parameter: 0.01 (4)

### Special details

**Table:**

Geometry. All e.s.d.'s (except the e.s.d. in the dihedral angle between two l.s. planes) are estimated using the full covariance matrix. The cell e.s.d.'s are taken into account individually in the estimation of e.s.d.'s in distances, angles and torsion angles; correlations between e.s.d.'s in cell parameters are only used when they are defined by crystal symmetry. An approximate (isotropic) treatment of cell e.s.d.'s is used for estimating e.s.d.'s involving l.s. planes.
Refinement. Refinement of *F*^2^ against ALL reflections. The weighted *R*-factor *wR* and goodness of fit *S* are based on *F*^2^, conventional *R*-factors *R* are based on *F*, with *F* set to zero for negative *F*^2^. The threshold expression of *F*^2^ > σ(*F*^2^) is used only for calculating *R*-factors(gt) *etc*. and is not relevant to the choice of reflections for refinement. *R*-factors based on *F*^2^ are statistically about twice as large as those based on *F*, and *R*- factors based on ALL data will be even larger.

### Fractional atomic coordinates and isotropic or equivalent isotropic displacement parameters (Å^2^)

**Table:**

	*x*	*y*	*z*	*U*_iso_*/*U*_eq_	
S	0.77287 (3)	0.499255 (18)	0.576519 (12)	0.01308 (5)	
O1	0.26383 (10)	0.24453 (5)	0.58832 (5)	0.01963 (14)	
O2	0.29792 (10)	0.39044 (5)	0.68639 (4)	0.01622 (12)	
O3	0.95092 (9)	0.44220 (6)	0.58061 (5)	0.02115 (14)	
O4	0.70626 (11)	0.56695 (6)	0.64934 (4)	0.02111 (14)	
C1	0.31824 (12)	0.34256 (7)	0.60812 (5)	0.01252 (14)	
C2	0.42632 (12)	0.42436 (7)	0.54745 (5)	0.01322 (14)	
H2	0.4070	0.5085	0.5662	0.016*	
C3	0.36297 (14)	0.41083 (10)	0.45331 (6)	0.0239 (2)	
H3A	0.4314	0.4668	0.4164	0.036*	
H3B	0.2295	0.4275	0.4493	0.036*	
H3C	0.3872	0.3295	0.4336	0.036*	
C4	0.19070 (16)	0.32121 (10)	0.74852 (7)	0.0258 (2)	
H4A	0.0614	0.3160	0.7288	0.039*	
H4B	0.1950	0.3598	0.8058	0.039*	
H4C	0.2433	0.2411	0.7529	0.039*	
C5	0.77229 (12)	0.59314 (7)	0.48448 (5)	0.01268 (14)	
C6	0.69566 (12)	0.70659 (7)	0.48923 (5)	0.01463 (14)	
H6	0.6427	0.7348	0.5421	0.018*	
C7	0.69813 (12)	0.77808 (7)	0.41487 (6)	0.01543 (15)	
H7	0.6459	0.8555	0.4174	0.019*	
C8	0.77573 (13)	0.73814 (7)	0.33690 (5)	0.01487 (15)	
C9	0.84746 (13)	0.62251 (8)	0.33331 (6)	0.01649 (16)	
H9	0.8974	0.5933	0.2801	0.020*	
C10	0.84653 (12)	0.55006 (7)	0.40655 (6)	0.01525 (15)	
H10	0.8959	0.4719	0.4037	0.018*	
C11	0.78446 (17)	0.81761 (9)	0.25811 (6)	0.02262 (18)	
H11A	0.6738	0.8683	0.2564	0.034*	
H11B	0.7893	0.7689	0.2052	0.034*	
H11C	0.8960	0.8674	0.2614	0.034*	
N	0.62422 (10)	0.39329 (6)	0.55827 (5)	0.01262 (13)	
H01	0.661 (2)	0.3437 (14)	0.5189 (10)	0.032 (4)*	

### Atomic displacement parameters (Å^2^)

**Table:**

	*U*^11^	*U*^22^	*U*^33^	*U*^12^	*U*^13^	*U*^23^
S	0.01306 (9)	0.01406 (8)	0.01212 (8)	−0.00207 (7)	−0.00239 (6)	0.00121 (7)
O1	0.0243 (3)	0.0136 (3)	0.0210 (3)	−0.0038 (2)	−0.0008 (3)	−0.0026 (2)
O2	0.0175 (3)	0.0183 (3)	0.0129 (3)	−0.0021 (2)	0.0007 (2)	−0.0013 (2)
O3	0.0131 (3)	0.0248 (3)	0.0256 (3)	−0.0009 (2)	−0.0058 (3)	0.0072 (3)
O4	0.0310 (4)	0.0199 (3)	0.0125 (3)	−0.0053 (3)	0.0004 (3)	−0.0036 (2)
C1	0.0109 (3)	0.0130 (3)	0.0137 (3)	0.0008 (3)	−0.0016 (3)	0.0000 (3)
C2	0.0113 (3)	0.0137 (3)	0.0146 (3)	0.0008 (3)	−0.0005 (3)	0.0020 (3)
C3	0.0167 (4)	0.0388 (5)	0.0163 (4)	−0.0023 (4)	−0.0046 (3)	0.0078 (4)
C4	0.0269 (5)	0.0319 (5)	0.0187 (4)	−0.0047 (4)	0.0050 (4)	0.0060 (4)
C5	0.0123 (3)	0.0128 (3)	0.0129 (3)	−0.0013 (3)	−0.0004 (3)	0.0003 (2)
C6	0.0141 (3)	0.0140 (3)	0.0158 (3)	0.0005 (3)	0.0016 (3)	−0.0013 (3)
C7	0.0145 (3)	0.0128 (3)	0.0190 (4)	0.0006 (3)	−0.0003 (3)	0.0004 (3)
C8	0.0150 (3)	0.0153 (3)	0.0143 (3)	−0.0032 (3)	−0.0031 (3)	0.0019 (3)
C9	0.0194 (4)	0.0164 (4)	0.0137 (4)	−0.0014 (3)	0.0022 (3)	−0.0011 (3)
C10	0.0167 (4)	0.0129 (3)	0.0162 (4)	0.0009 (3)	0.0014 (3)	−0.0012 (3)
C11	0.0299 (5)	0.0199 (4)	0.0180 (4)	−0.0044 (4)	−0.0027 (4)	0.0050 (3)
N	0.0112 (3)	0.0109 (3)	0.0157 (3)	0.0003 (2)	−0.0002 (2)	−0.0002 (2)

### Geometric parameters (Å, °)

**Table:**

S—O4	1.4341 (7)	C9—C10	1.3878 (12)
S—O3	1.4344 (7)	C2—H2	1.0000
S—N	1.6262 (8)	C3—H3A	0.9800
S—C5	1.7628 (8)	C3—H3B	0.9800
O1—C1	1.2094 (10)	C3—H3C	0.9800
O2—C1	1.3235 (10)	C4—H4A	0.9800
O2—C4	1.4525 (11)	C4—H4B	0.9800
C1—C2	1.5223 (11)	C4—H4C	0.9800
C2—N	1.4755 (11)	C6—H6	0.9500
C2—C3	1.5213 (13)	C7—H7	0.9500
C5—C6	1.3929 (11)	C9—H9	0.9500
C5—C10	1.3956 (11)	C10—H10	0.9500
C6—C7	1.3955 (11)	C11—H11A	0.9800
C7—C8	1.3938 (12)	C11—H11B	0.9800
C8—C9	1.4012 (12)	C11—H11C	0.9800
C8—C11	1.5043 (12)	N—H01	0.865 (16)
			
O4—S—O3	120.15 (5)	C2—C3—H3B	109.5
O4—S—N	107.69 (4)	H3A—C3—H3B	109.5
O3—S—N	105.46 (4)	C2—C3—H3C	109.5
O4—S—C5	107.69 (4)	H3A—C3—H3C	109.5
O3—S—C5	107.79 (4)	H3B—C3—H3C	109.5
N—S—C5	107.48 (4)	O2—C4—H4A	109.5
C1—O2—C4	115.78 (7)	O2—C4—H4B	109.5
O1—C1—O2	124.28 (8)	H4A—C4—H4B	109.5
O1—C1—C2	124.32 (8)	O2—C4—H4C	109.5
O2—C1—C2	111.35 (7)	H4A—C4—H4C	109.5
N—C2—C3	111.84 (7)	H4B—C4—H4C	109.5
N—C2—C1	106.32 (7)	C5—C6—H6	120.6
C3—C2—C1	111.48 (7)	C7—C6—H6	120.6
C6—C5—C10	120.98 (7)	C8—C7—H7	119.3
C6—C5—S	120.57 (6)	C6—C7—H7	119.3
C10—C5—S	118.43 (6)	C10—C9—H9	119.6
C5—C6—C7	118.74 (7)	C8—C9—H9	119.6
C8—C7—C6	121.32 (7)	C9—C10—H10	120.3
C7—C8—C9	118.74 (7)	C5—C10—H10	120.3
C7—C8—C11	120.90 (8)	C8—C11—H11A	109.5
C9—C8—C11	120.36 (8)	C8—C11—H11B	109.5
C10—C9—C8	120.81 (8)	H11A—C11—H11B	109.5
C9—C10—C5	119.37 (8)	C8—C11—H11C	109.5
C2—N—S	118.70 (6)	H11A—C11—H11C	109.5
N—C2—H2	109.0	H11B—C11—H11C	109.5
C3—C2—H2	109.0	C2—N—H01	111.8 (10)
C1—C2—H2	109.0	S—N—H01	113.1 (10)
C2—C3—H3A	109.5		
			
C4—O2—C1—O1	−4.64 (13)	C5—C6—C7—C8	−0.17 (13)
C4—O2—C1—C2	177.89 (8)	C6—C7—C8—C9	1.82 (13)
O1—C1—C2—N	−87.54 (10)	C6—C7—C8—C11	−177.58 (9)
O2—C1—C2—N	89.93 (8)	C7—C8—C9—C10	−1.88 (13)
O1—C1—C2—C3	34.58 (12)	C11—C8—C9—C10	177.52 (9)
O2—C1—C2—C3	−147.95 (8)	C8—C9—C10—C5	0.29 (13)
O4—S—C5—C6	−6.70 (9)	C6—C5—C10—C9	1.42 (13)
O3—S—C5—C6	−137.70 (7)	S—C5—C10—C9	−179.99 (7)
N—S—C5—C6	109.07 (7)	C3—C2—N—S	108.25 (8)
O4—S—C5—C10	174.70 (7)	C1—C2—N—S	−129.86 (6)
O3—S—C5—C10	43.70 (8)	O4—S—N—C2	52.67 (7)
N—S—C5—C10	−69.53 (8)	O3—S—N—C2	−177.90 (6)
C10—C5—C6—C7	−1.48 (13)	C5—S—N—C2	−63.10 (7)
S—C5—C6—C7	179.97 (6)		

### Hydrogen-bond geometry (Å, °)

**Table:**

*D*—H···*A*	*D*—H	H···*A*	*D*···*A*	*D*—H···*A*
N—H01···O1^i^	0.865 (16)	2.057 (16)	2.9097 (10)	168.6 (14)
C10—H10···O1^i^	0.95	2.62	3.3696 (11)	136
C9—H9···O2^ii^	0.95	2.63	3.4065 (11)	140
C7—H7···O3^iii^	0.95	2.67	3.6167 (11)	172
C4—H4C···O4^iv^	0.98	2.49	3.3453 (13)	145

Symmetry codes: (i) *x*+1/2, −*y*+1/2, −*z*+1; (ii) −*x*+3/2, −*y*+1, *z*−1/2; (iii) *x*−1/2, −*y*+3/2, −*z*+1; (iv) −*x*+1, *y*−1/2, −*z*+3/2.
